# Hepatitis B Core‐Related Antigen Level Predicts Disease Progression in the Gray Zone Hepatitis B Patients

**DOI:** 10.1002/jmv.70925

**Published:** 2026-04-08

**Authors:** Takanori Suzuki, Kentaro Matsuura, Takako Inoue, Hayato Kawamura, Kei Fujiwara, Hiromi Kataoka, Yasuhito Tanaka

**Affiliations:** ^1^ Department of Gastroenterology and Metabolism Nagoya City University Graduate School of Medical Sciences Nagoya Japan; ^2^ Department of Clinical Laboratory Medicine Nagoya City University Hospital Nagoya Japan; ^3^ Department of Gastroenterology and Hepatology Kumamoto University Kumamoto Japan

**Keywords:** chronic hepatitis B, gray zone, hepatitis B core‐related antigen

## Abstract

**Objectives:**

The natural history of hepatitis B virus (HBV) infection does not always conform to the currently defined phases, and some patients who do not clearly meet established treatment criteria are classified as in the gray zone (GZ). We investigated the factors for progression of hepatitis B e antigen (HBeAg)‐negative GZ patients to chronic hepatitis (CH): HBV DNA levels ≥ 3.3 log IU/mL and alanine aminotransferase (ALT) ≥ 31 U/L.

**Methods:**

This study included 102 HBeAg‐negative GZ patients (81 with high HBV DNA levels and normal ALT levels, and 21 with low HBV DNA levels and high ALT levels), and who underwent liver stiff measurement using transient elastography.

**Results:**

Of 91 patients followed up for more than 6 months, 19 progressed to CH, and the cumulative incidence rate of progression to CH at 5 years was 17.5%. Multivariate analysis showed that only the hepatitis B core‐related antigen (HBcrAg) level was independently associated with progression to CH (HR 3.08; *p* = 0.003). Receiver operating characteristic curve analysis to assess the effectiveness of HBcrAg levels in predicting progression to CH showed the area under the curve was 0.828, and the optimal cut‐off value was determined to be 2.87 log U/mL. When applying this cut‐off, the cumulative incidence rates of progression to CH were significantly divided (*p* < 0.001): 5‐year rates were 39.1% in patients with HBcrAg levels ≥ 2.87 log U/mL and 2.9% in those with levels < 2.87 log U/mL.

**Conclusions:**

HBcrAg levels are useful for predicting progression to CH by HBeAg‐negative GZ patients.

**Clinical Trial Registration:**

The study protocol was approved by the Institutional Review Board of Nagoya City University (approval number: 60‐00‐0657).

**Abbreviations**
ALT: Alanine transaminase
AST: Aspartate aminotransferase
CHB: Chronic hepatitis B
DM: Diabetes mellitus
FIB‐4: Fibrosis‐4
GZ: Gray zone
HBV: Hepatitis B virus
HCC: Hepatocellular carcinoma
HCV: Hepatitis C virus
HIV: Human immunodeficiency virus
IC: Inactive carrier
NA: Nucleot(s)ide analogues

## 1 Introduction

Persistent hepatitis B virus (HBV) infection remains a significant global health concern, with recent estimates suggesting that over 292 million people are positive for hepatitis B surface antigen (HBsAg) [1]. Individuals with chronic hepatitis B (CHB) have an elevated risk of developing serious complications, including hepatocellular carcinoma (HCC) [2].

From the clinical perspective, HBV infection does not always conform to the currently defined phases of its natural history, and some patients do not clearly meet established treatment criteria. For instance, certain individuals may have HBV DNA ≥ 2,000 IU/mL while maintaining ‘normal’ alanine transaminase (ALT) values, whereas others may have HBV DNA < 2,000 IU/mL but elevated ALT levels [3]. Patients who fall into either of these groups and decline a diagnostic liver biopsy to determine the extent of fibrosis are categorized as being in an ‘indeterminate phase’ or ‘gray zone (GZ)’ [4]. Recent evidence indicates that as many as 30%–50% of patients with chronic HBV infection may fall into this category [5, 6, 7]. Therefore, a substantial subset of HBV patients does not fit neatly into the established disease stages and is instead categorized as being in a GZ, a state with no definitive therapeutic indication. The risk of HCC development in patients in this indeterminate phase, and who are not eligible for anti‐HBV treatment, is known to be higher than that in inactive carriers (IC) [5]. Meanwhile, it has been reported that patients who have transitioned from GZ to IC do not progress to liver fibrosis and liver cirrhosis, which is the main cause of HCC [8]. Therefore, it is important to identify the factors that make it more likely for GZ patients to progress to chronic hepatitis (CH), which requires treatment with antiviral agents.

A newly developed, fully automated, high‐sensitivity chemiluminescent enzyme immunoassay (CLEIA), known as iTACT‐HBcrAg (Fujirebio Inc., Tokyo, Japan), provides approximately ten times greater sensitivity than the conventional HBcrAg assay. Several studies have shown that iTACT‐HBcrAg is useful for monitoring patients and for the early detection of HBV reactivation [9, 10]. Furthermore, recently, its usefulness for predicting HCC development in hepatitis B e antigen (HBeAg)‐negative GZ patients also has been reported [11]. However, its usefulness for predicting the progression to CH in HBeAg‐negative GZ patients has not yet been adequately investigated.

Therefore, a retrospective investigation was carried out into the factors associated with progression to CH in HBeAg‐negative GZ patients.

## 2 Patients and Methods

### 2.1 Patients

**Figure 1:**
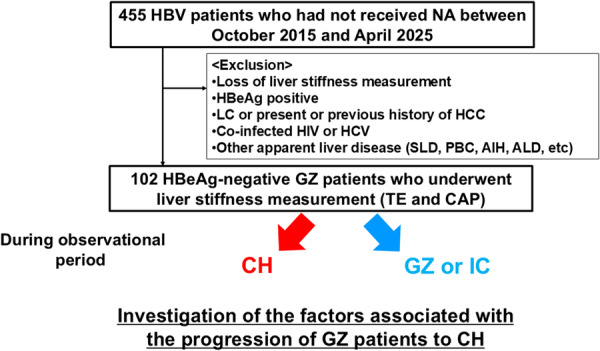
Flow chart of this study. Abbreviations: AIH, autoimmune hepatitis; ALD, alcohol‐related liver disease; CAP, controlled attenuation parameter; CH, chronic hepatitis; GZ, gray zone; HBV, hepatitis B virus; HBeAg, hepatitis B e antigen; HCC, hepatocellular carcinoma; HCV, hepatitis C virus; HIV, human immunodeficiency virus; IC, inactive carrier; LC, liver cirrhosis; NA, nucleot(s)ide analogue; PBC, primary biliary cholangitis; SLD, steatotic liver disease; TE, transient elastography.

A flowchart of this study is shown in Figure 1. A total of 455 HBV patients, who had not received nucleot(s)ide analogues (NA) previously, presented to Nagoya City University Hospital between October 2015 and April 2025. The following were excluded from the study: patients who had not undergone liver stiffness measurement, those who were HBeAg positive, and those with clinically diagnosed liver cirrhosis or a prior history of HCC. None of the patients included in the study were co‐infected with human immunodeficiency virus or hepatitis C virus, nor did they have other chronic liver diseases, such as autoimmune hepatitis or primary biliary cholangitis. We also excluded patients with apparent steatotic liver disease or alcohol‐related liver disease diagnosed at baseline and during the follow‐up by attending physicians. Consequently, this study included 102 HBeAg‐negative GZ hepatitis B patients who underwent liver stiffness measurement using transient elastography (TE) by FibroScan® (Echosens, Paris, France). All of the 102 study patients failed to fulfil the criteria of the Japan Society of Hepatology Guidelines for the Management of HBV Infection to be eligible for anti‐HBV therapy, with HBV DNA levels ≥ 3.3 log IU/mL and ALT ≥ 31 U/L [12]. We defined the GZ patients as follows: those who had high HBV DNA levels ≥ 3.3 log IU/mL and ALT < 31 U/L (DNA‐H group, *n* = 81), and those who had low HBV DNA levels < 3.3 log IU/mL and ALT ≥ 31 U/L (DNA‐L group, *n* = 21). The patients were monitored approximately every 6 months. The observational period commenced on the day TE was performed and ended the day of the most recent visit.

### 2.2 Baseline Data of the Study Patients, Laboratory and Histological Tests and Liver Stiffness Measurements

Demographic data were obtained as follows: age, sex, blood pressure, history of type 2 diabetes mellitus (DM) or specific treatment for DM, hyperlipidemia (hyperlipidemia or antihyperlipidemic treatment), hypertension (high systolic blood pressure or antihypertensive treatment).

Serum samples stored at –80°C were analyzed for HBcrAg using the iTACT‐HBcrAg assay. The lower limit of quantification (LLOQ) for the iTACT‐HBcrAg assays is 2.1 log U/mL [9]. In this study, iTACT‐HBcrAg levels less than 2.1 log U/mL were recorded as 2.0 log U/mL. HBsAg concentrations were mostly determined with the conventional HISCL HBsAg assay (Sysmex Corporation, Kobe, Japan), which has an LLOQ of 0.03 IU/mL; in a small number of cases, HBsAg was quantified using other standardized methods. Serum HBeAg levels were assessed using an automated CLEIA platform (HISCL HBeAg; Sysmex Corporation, Kobe, Japan) with a cutoff index of 1.0. HBV DNA was quantified with the COBAS AmpliPrep/COBAS TaqMan HBV v2.0 assay (Roche Diagnostics K.K., Tokyo, Japan) according to the manufacturer's protocol. Up to 2017, the LLOQ was 2.1 log copies/mL with an upper limit of 9.0 log copies/mL; after 2017, these thresholds were revised to 1.3 log IU/mL and 8.2 log IU/mL, respectively. The manufacturer's recommended conversion was applied (log IU/mL = log copies/mL – 0.76). Additional hematologic and biochemical parameters were evaluated using routine clinical assays.

The histological hepatic fibrosis index, Fibrosis‐4 (FIB‐4) index, was calculated as described previously: FIB‐4 = (age [year] × aspartate aminotransferase (AST) [U/L])/(platelet count [10^9/^L] × √ALT [U/L]) [13].

FibroScan assessments were conducted using the FibroTouch 502 device (Echosens, Paris, France). All examinations followed the manufacturer's instructions. In addition, the controlled attenuation parameter (CAP), which reflects the degree of hepatic steatosis, was measured for each patient during the FibroScan procedure.

### 2.3 Definitions of Chronic Hepatitis and Inactive Carrier, and Primary Outcome

Referring to the Japan Society of Hepatology Guidelines for the Management of HBV Infection, patients who needed anti‐HBV therapy, with HBV DNA levels ≥ 3.3 log IU/mL and ALT ≥ 31 U/L were defined as CH [12]. IC was defined as follows: being HBeAg‐negative, hepatitis B e antibody (anti‐HBe)‐positive, HBV DNA < 3.3 log IU/ml, and ALT < 31 IU/L. During the follow‐up, once the diagnostic criteria for CH had been met, NAs were usually initiated by the attending physicians. As a result, some patients were determined to meet the CH criteria on the basis of two consecutive visits. Therefore, progression from GZ to CH, the primary outcome of this study, was defined as cases in which both HBV DNA ≥ 3.3 log IU/mL and ALT ≥ 31 U/L were temporarily observed. Meanwhile, even after GZ patients achieved IC status, subsequent progression to CH was assessed up to the final observation period.

### 2.4 Ethical Standards

The study protocol was approved by the Institutional Review Board of Nagoya City University (approval number: 60‐00‐0657).

### 2.5 Statistical Analysis

Categorical variables are presented as numbers, while continuous variables were expressed as medians with interquartile ranges (IQRs). Categorical variables were compared using the chi‐square test, whereas non‐categorical variables were compared using the Mann–Whitney *U* test. The cumulative incidence of progression to CH was estimated by the Kaplan–Meier method, and the log‐rank test was employed to assess differences in cumulative incidence. Univariate and multivariate analyses to determine factors associated with progression to CH (HBV DNA levels ≥ 3.3 log IU/mL and ALT ≥ 31 U/L) were performed using the stepwise Cox proportional hazards model. Receiver operating characteristic (ROC) curve analysis was used to calculate the area under the curve (AUC), assessing the effectiveness of HBcrAg levels in distinguishing the progression to CH. ROC analysis was also used to identify the optimal cut‐off value with the maximum sensitivity and specificity for differentiating the specified groups. In this study, data regarding HBcrAg were available only for a subset of patients. Missing HBcrAg data were treated as absent and were not inputted. Accordingly, all analyses involving HBcrAg data were performed using complete‐case analyses. Statistical significance was defined as a *p*‐value < 0.05. All analyses were carried out with EZR (Easy R, Saitama Medical Center, Jichi Medical University, Saitama, Japan), a modified distribution of R Commander (version 1.61) [14].

## 3 Results

### 3.1 Characteristics of the Study Population

All of the patients were assigned to the DNA‐H group (*n* = 81) or the DNA‐L group (*n* = 21). The baseline clinical characteristics of the patients in these two groups are shown in Table 1. Comparing the two groups, the DNA‐H group had significantly lower aspartate aminotransferase (AST), ALT, and FIB‐4 index than DNA‐L group (*p* < 0.001, < 0.001, and 0.025), as well as significantly lower TE and CAP values (*p* = 0.003 and < 0.001), and significantly higher HBV DNA levels (*p *< 0.001). Of the 102 study patients, HBcrAg measurement using stored serum was possible in 76, and there was no difference in HBcrAg levels between the two groups. In addition, we compared the backgrounds of the study patients between those with and without HBcrAg data. These results are summarized in Supplementary Table 1. The study patients for whom HBcrAg data were available had significantly lower platelet counts than those lacking those data.

**Table 1:** Baseline clinical characteristics of the 102 HBeAg‐negative GZ patients.

Number (*n* = 102)	DNA‐H group (*n* = 81)	DNA‐L group (*n* = 21)	*p* value
Age, years	48 (41–59)	52 (43–58)	0.616
Gender, male/female	30/51	11/10	0.221
Hypertension, yes/no	7/74	4/17	0.231
Diabetes, yes/no	15/66	2/19	0.513
Hyperlipidemia, yes/no	10/71	4/17	0.479
Platelet count (× 10^4^/μL)	21.4 (18.6–24.5)	20.9 (17.5–25.5)	0.911
AST (U/L)	21 (18–24)	38 (30–54)	< 0.001**
ALT (U/L)	18 (15–21)	46 (33–66)	< 0.001**
Albumin (mg/dL)	4.5 (4.3–4.7)	4.4 (4.4–4.7)	0.451
Total bilirubin (mg/dL)	0.8 (0.7–1.0)	0.7 (0.6–0.9)	0.127
FIB‐4 index	1.06 (0.86–1.47)	1.45 (1.24–1.83)	0.025*
HBV DNA level (log IU/mL)	3.8 (3.4–4.4)	2.1 (1.6–2.8)	< 0.001**
HBcrAg (log U/mL)	2.5 (2.0–3.2)	2.1 (2.0–3.0)	0.720
HBsAg level (IU/mL)	1067.21 (337.41–2824.71)	226.69 (17.48–1981.56)	0.043*
Time from TE to the last visit, months	89 (60–103)	57 (24–88)	0.067
TE, kPa	3.8 (3.3–4.9)	5.3 (4.3–5.9)	0.003**
CAP, dm/mm	215 (186–228)	272 (231–321)	< 0.001**

*Note:* DNA‐H group: HBeAg‐negative GZ patients who had high HBV DNA levels ≥ 3.3 log IU/mL and ALT < 31 U/L. DNA‐L group: HBeAg‐negative GZ patients who had low HBV DNA levels < 3.3 log IU/mL and ALT ≥ 31 U/L. Data are expressed as numbers for categorical data and medians (first–third quartiles) for non‐categorical data, and compared using the chi‐square test and the Mann–Whitney *U* test, respectively.

Abbreviations: ALT, alanine aminotransferase; AST, aspartate aminotransferase; CAP, controlled attenuation parameter; FIB‐4, fibrosis‐4; GZ, gray zone; HBcrAg, hepatitis B core related antigen; HBeAg, hepatitis B e antigen; HBsAg, hepatitis B surface antigen; HBV, hepatitis B virus; TE, transient elastography.

* *p* < 0.05

** *p* < 0.005.

### 3.2 Cumulative Incidence of Progression to CH

The median observational period for all the study patients was 89 months (IQR: 63–103 months) and 91 (73 in DNA‐H group and 18 in DNA‐L group) were followed up for more than 6 months. Of the 73 patients in the DNA‐H group, 16 progressed to CH, 24 remained in the GZ, and 33 became IC during the observation period. On the other hand, of the 18 patients in DNA‐L group, 3 progressed to CH, 7 remained in the GZ, and 8 became IC (Supplementary Figure 1). Thus, 19 of the 91 patients progressed to CH. The cumulative incidence rate of progression to CH at 5 years after the start of follow‐up was 17.5% (Supplementary Figure 2).

### 3.3 Factors Associated With Progression to CH

Univariate and multivariate analyses were conducted using the Cox proportional hazards model to determine the factors associated with progression to CH. In the univariate analysis, significant favorable predictors were lower age, and higher levels of HBV DNA and HBcrAg. When these factors were entered into the multivariate analysis, only a higher HBcrAg level (HR 3.08; *p *= 0.003) was independently associated with progression to CH (Table 2).

**Table 2:** Factors associated with progressing to CH in HBeAg‐negative GZ patients.

Factor	Univariate analysis		Multivariate analysis	
SD (*n* =62)	HR (95% CI)	*p* value	HR (95% CI)	*p* value
Age (per 1 years)	0.92 (0.88–0.97)	< 0.001**	0.94 (0.89–1.00)	0.055
Gender (female/male)	2.23 (0.90–5.55)	0.085		
Presence of hypertension (yes/no)	1.35 (0.49–3.77)	0.562		
Presence of diabetes (yes/no)	0.50 (0.07–3.72)	0.495		
Presence of hyperlipidemia (yes/no)	0.61 (0.14–2.65)	0.511		
Platelet count (per 1 × 10^4^/μL)	1.00 (0.99–1.01)	0.375		
AST (per 1 U/L)	1.00 (0.98–1.02)	0.947		
ALT (per 1 U/L)	1.00 (0.99–1.01)	0.618		
Albumin (per 1 mg/dL)	0.52 (0.09–2.92)	0.457		
Total bilirubin (per 1 mg/dL)	0.27 (0.04–1.78)	0.174		
HBV DNA (per 1 log IU/mL)	1.85 (1.02–3.37)	0.044*	1.41 (0.68–2.91)	0.358
HBcrAg (per 1 log U/mL)	4.25 (2.24–8.07)	< 0.001**	3.08 (1.47–6.49)	0.003**
HBsAg (per 1 IU/mL)	1.00 (1.00–1.00)	0.464		
TE, per 1 kPa	1.04 (0.86–1.26)	0.697		
CAP, per 1 dm/mm	1.00 (0.99–1.01)	0.376		

*Note:* Hazard ratios were calculated using the Cox proportional hazard method.

Abbreviations: ALT, alanine aminotransferase; AST, aspartate aminotransferase; CAP, controlled attenuation parameter; CH, chronic hepatitis; CI, confidence interval; GZ, gray zone; HBcrAg, hepatitis B core related antigen; HBeAg, hepatitis B e antigen; HBsAg, hepatitis B surface antigen; HBV, hepatitis B virus; HR, hazard ratio; TE, transient elastography.

* *p* < 0.05

** *p* < 0.005.

### 3.4 Optimal Cut‐Off Value of HBcrAg for Predicting Progression to CH

**Figure 2:**
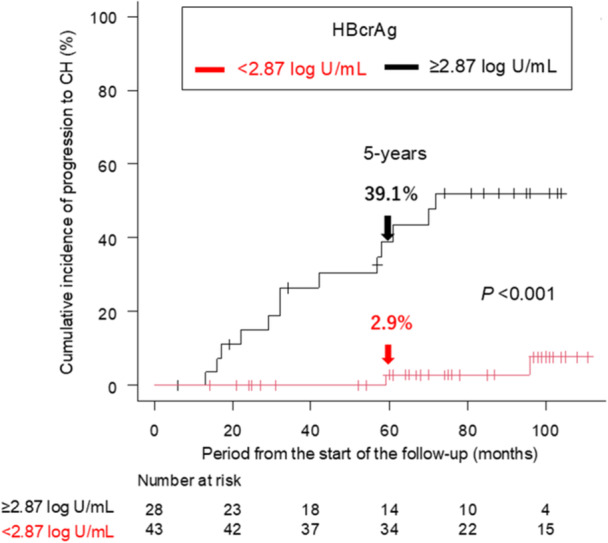
Cumulative incidence rates of progression to CH of HBeAg‐negative GZ patients, stratified by HBcrAg levels. *p*‐values were calculated using log‐rank testing. Abbreviations: CH, chronic hepatitis; GZ, gray zone; HBcrAg, hepatitis B core‐related antigen; HBeAg, hepatitis B e antigen.

We carried out ROC curve analysis to assess the effectiveness of HBcrAg levels in distinguishing the progression to CH in the 71 study patients who were followed up for more than 6 months and whose serum samples were available for HBcrAg measurement. The AUC of HBcrAg for dividing these patients into those who progressed to CH and those who did not was 0.828, and the optimal cut‐off value was determined to be 2.87 log U/mL (sensitivity = 0.732, specificity = 0.867, Supplementary Figure 3). When applying this HBcrAg cut‐off, the cumulative incidence rates of progression to CH were significantly separated (*p* < 0.001): the 5‐year rates were 39.1% in the patients with ≥ 2.87 log U/mL and 2.9% in those with < 2.87 log U/mL (Figure 2).

### 3.5 Transition of HBV DNA Levels in DNA‐H and ‐L Patients

**Figure 3:**
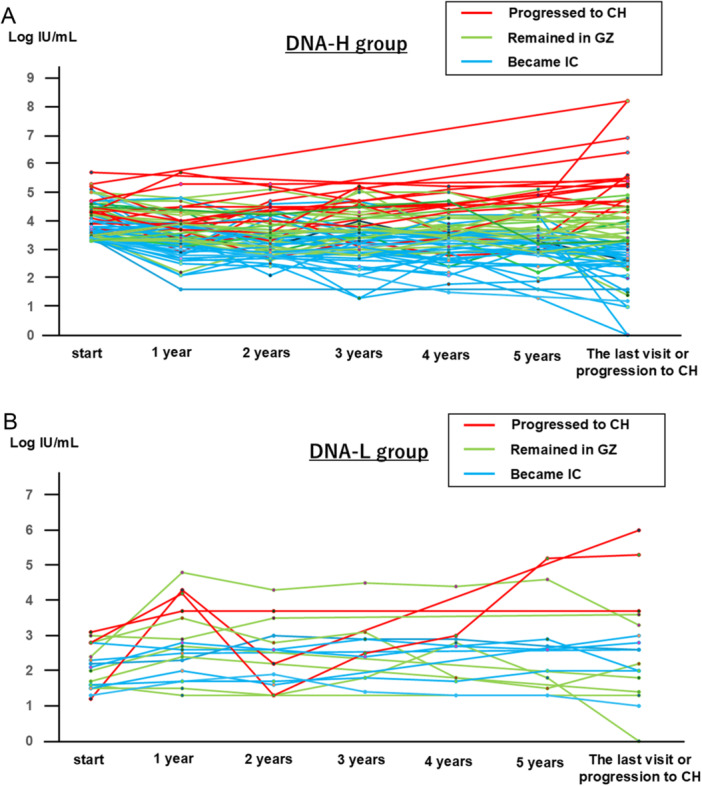
Transition of HBV DNA levels in HBeAg‐negative GZ patient. (A) Transition of HBV DNA levels in DNA‐H group (*n* = 73): HBeAg‐negative GZ patients who had high HBV DNA levels ≥ 3.3 log IU/mL and ALT < 31 U/L. (B) Transition of HBV DNA levels in DNA‐L group (*n* = 18): HBeAg‐negative GZ patients who had low HBV DNA levels < 3.3 log IU/mL and ALT ≥ 31 U/L. Red, green, and blue lines represent the transition of HBV DNA in the patients who progressed to CH, remained in the GZ, and became IC during the observational period, respectively. ALT, alanine aminotransferase; CH, chronic hepatitis; GZ, gray zone; HBV, hepatitis B virus; HBeAg, hepatitis B e antigen; IC, inactive carrier.

Figures 3A and 3B show the transition of HBV DNA levels in the DNA‐H and DNA‐L cases, respectively. In both groups, the patients who progressed to CH had elevated HBV DNA levels prior to progressing to CH (red line), while the cases who became IC had low HBV DNA levels during the observational period (blue line).

## 4 Discussion

This study investigated the factors associated with the progression of HBeAg‐negative GZ patients to CH. The cumulative incidence rate of progression to CH was 17.5% at 5 years (Supplementary Figure 2). We identified the HBcrAg level as a significant factor for predicting progression to CH (Table 2), and the optimal cut‐off value of HBcrAg significantly stratified the cumulative incidence rates of progression to CH (Figure 2). A previous study showed that the HBcrAg level, rather than the HBV DNA level, was a predictive factor for HCC development in HBeAg‐negative GZ patients [11]. However, its usefulness for predicting the progression of HBeAg‐negative GZ patients to CH had not been investigated previously. We believe this is the first study to show that HBcrAg levels contribute to progression from HBeAg‐negative GZ to CH.

In this study, we first divided the HBeAg‐negative GZ patients into the two groups, based on their HBV DNA and ALT levels (DNA‐H and DNA‐L groups), and compared their backgrounds. We found that the patients in DNA‐L group had higher TE and CAP values. The results of this study are consistent with a report that borderline ALT elevation, accompanied by low HBV DNA levels, may indicate either ongoing necroinflammatory activity or the presence of concomitant steatotic liver disease [15].

The univariate analyses showed that younger age and higher levels of HBV DNA and HBcrAg are factors associated with the progression of HBeAg‐negative GZ patients to CH, but the multivariate analysis revealed that only HBcrAg was a significant factor (Table 2). HBcrAg consists of three proteins encoded by the precore/core region: HBeAg, hepatitis B core antigen (HBcAg), a component of the virion, and a 22 kDa precore protein (p22cr), which is a component of HBV DNA‐negative (empty) particles [16, 17]. HBcrAg has been used for monitoring the clinical course of HBV‐infected patients and to determine the patients' outcomes [18, 19]. In particular, serum HBcrAg levels correlate with intrahepatic covalently closed circular DNA (cccDNA) levels, as well as with serum HBV DNA levels, which provide indicators of HBV replication [20, 21, 22, 23]. Therefore, we assume that HBeAg‐negative GZ patients with high HBcrAg levels are more likely to progress to CH. As mentioned in the introduction, the highly sensitive iTACT‐HBcrAg assay (LLOQ: 2.1 log U/mL) has been established and is now used in clinical practice [9]. The optimal cut‐off value of HBcrAg for predicting progression to CH was 2.87 log U/mL in this study, which is below the LLOQ of the conventional HBcrAg assay. Critically, this highly sensitive iTACT‐HBcrAg assay has revealed the clinical significance of HBcrAg levels for determining the prognosis of GZ patients.

Furthermore, we demonstrated that HBeAg‐negative GZ cases with persistently high HBV DNA levels tended to progress to CH (Figures 3A and 3B). We think this result is reasonable because the HBV DNA level is an indicator of HBV activity, in addition to HBcrAg.

Previous studies revealed that the risk of HCC development in patients in the indeterminate phase was higher than in IC patients [5], and anti‐HBV therapy reduced the risk of HCC of patients in the indeterminate phase [24]. Therefore, it is considered preferable to administer anti‐HBV therapy, particularly for GZ patients transitioning to CH. Our results indicate that HBcrAg levels and HBV DNA trends during follow‐up are useful for predicting the progression to CH and consideration of the timing of administration of NAs to HBeAg‐negative GZ patients.

Based on the results of the present study, HBeAg‐negative GZ patients with high HBcrAg levels have a high rate of progression to CH. Therefore, one possible management strategy is to reduce the follow‐up interval for such patients and promptly initiate treatment with NAs when progression to CH is observed. Alternatively, it may be reasonable to be more proactive and consider initiating NA in HBeAg‐negative GZ patients with elevated HBcrAg levels. These treatment strategies require investigation in future studies. In addition, we conducted this study in a Japanese population and, therefore, followed the Japan Society of Hepatology Guidelines for the Management of HBV Infection [12]. Although international guidelines, such as those from the European Association for the Study of the Liver (EASL) and American Association for the Study of Liver Diseases (AASLD), use different ALT cut‐off values for the definition of CH, the overall concept is similar [25, 26]. Accordingly, HBcrAg is expected to be useful for predicting progression to CH, even in studies involving different populations and guideline definitions; however, further validation in diverse cohorts is warranted.

This study has several limitations. First, it was conducted as a retrospective, single‐center analysis with a relatively small sample size. In particular, the number of patients in the DNA‐L group was limited in our cohort, and this may have affected the robustness of the analysis. Furthermore, given the limited number who progressed to CH, multivariable Cox modelling may have led to overfitting. In addition, due to the small sample size, the lack of internal validation is acknowledged, and the proposed HBcrAg cut‐off is presented as exploratory, pending external validation. To validate our observations, future investigations should include larger, prospective cohorts with longer follow‐up durations. Second, there were potential occurrences of selection bias in this study. This study was conducted in a Japanese population, and whether the findings can be generalized to populations in other countries remains an issue for future investigation. In addition, the HBV genotype could not be measured in patients with low HBV DNA levels, and therefore could not be examined in this study. However, genotype C accounts for the vast majority of cases in Japan [27]. Furthermore, the study patients with available HBcrAg data had significantly lower platelet counts than those without such data. However, the association between HBcrAg levels and clinical outcomes remained stable after adjustment for platelet counts, indicating that the observed imbalance did not confound the primary findings (Table 2). Third, alternative causes of ALT elevation, such as steatotic or alcohol‐related disease, could not be completely excluded at progression to CH. The values of TE and CAP were not systematically available for all follow‐up time points. Future studies with serial TE and CAP assessment are warranted. Fourth, the analysis focused on a single evaluation of the HBcrAg level and longitudinal evaluation of HBcrAg levels may offer further clinical insight. Fifth, regular assessments of metabolic factors, such as HbA1c and other related laboratory parameters, as well as blood pressure measurement, were not available, so that longitudinal changes in metabolic factors could not be evaluated in this study. Sixth, cytokine and chemokine profiles related to immune response were not systematically assessed in this study. Further investigation of the association of these markers with disease progression is warranted in the future.

In summary, we found that the cumulative incidence of progression to CH was high in HBeAg‐negative GZ patients with high HBcrAg levels. The results of this study may be useful in consideration of the timing of administration of NAs to HBeAg‐negative GZ patients. However, additional prospective studies are required to confirm these findings.

## Author Contributions

Conception and design: T Suzuki, K Matsuura and Y Tanaka. drafting of manuscript: T Suzuki and K Matsuura. acquisition, analysis and interpretation of data: T Suzuki, K Matsuura, T Inoue, H Kawamura, K Fujiwara, H Kataoka, Y Tanaka. critical revision of the manuscript: K Matsuura, Y Tanaka. study supervision: Y Tanaka.

## Ethics Statement

The study was implemented according to the Declaration of Helsinki.

## Consent

Written informed consent was obtained from all the patients.

## Conflicts of Interest

Yasuhito Tanaka: Research funding from AbbVie GK., OTSUKA Pharmaceutical Co. Ltd., FUJIREBIO Inc. Sysmex Corp., GlaxoSmithKline PLC., Gilead Sciences Inc., AstraZeneca, and honoraria from AbbVie GK., Gilead Sciences Inc., Chugai Pharmaceutical Co. Ltd., ASKA Pharmaceutical Holdings Co. Ltd., GlaxoSmithKline PLC., AstraZeneca, Eisai, HU frontier Takako Inoue: Research funding from Fujirebio Inc and Sysmex Corporation. The other authors declare no conflicts of interest.

## Supporting information


Supporting File 1

Supporting File 2

## Data Availability

The authors confirm that the data supporting the findings of this study are available within the article.
