# Supplementary material for: Hepatitis B Core‐Related Antigen Level Predicts Disease Progression in the Gray Zone Hepatitis B Patients
Source: J Med Virol. 2026 Apr 8;98(4):e70925. doi: 10.1002/jmv.70925 (PMC13060282; doi:10.1002/jmv.70925)
Supplement: Supplementary file 1 — Supporting File 1 [file JMV-98-e70925-s001.docx]

**Supplementary Information**

Hepatitis B core-related antigen level predicts disease progression

in the gray zone hepatitis B patients

**Author:** Takanori Suzuki, Kentaro Matsuura, Takako Inoue, Hayato Kawamura,　Kei Fujiwara, Hiromi Kataoka, Yasuhito Tanaka

Supplementary Figure 1..……………………………………………………………...P2

Supplementary Figure 2.……………………………………………………………...P3

Supplementary Figure 3.……………………………………………………………...P4

Supplementary Table 1.……………………………………………………………...P5

Supplementary Figure 1


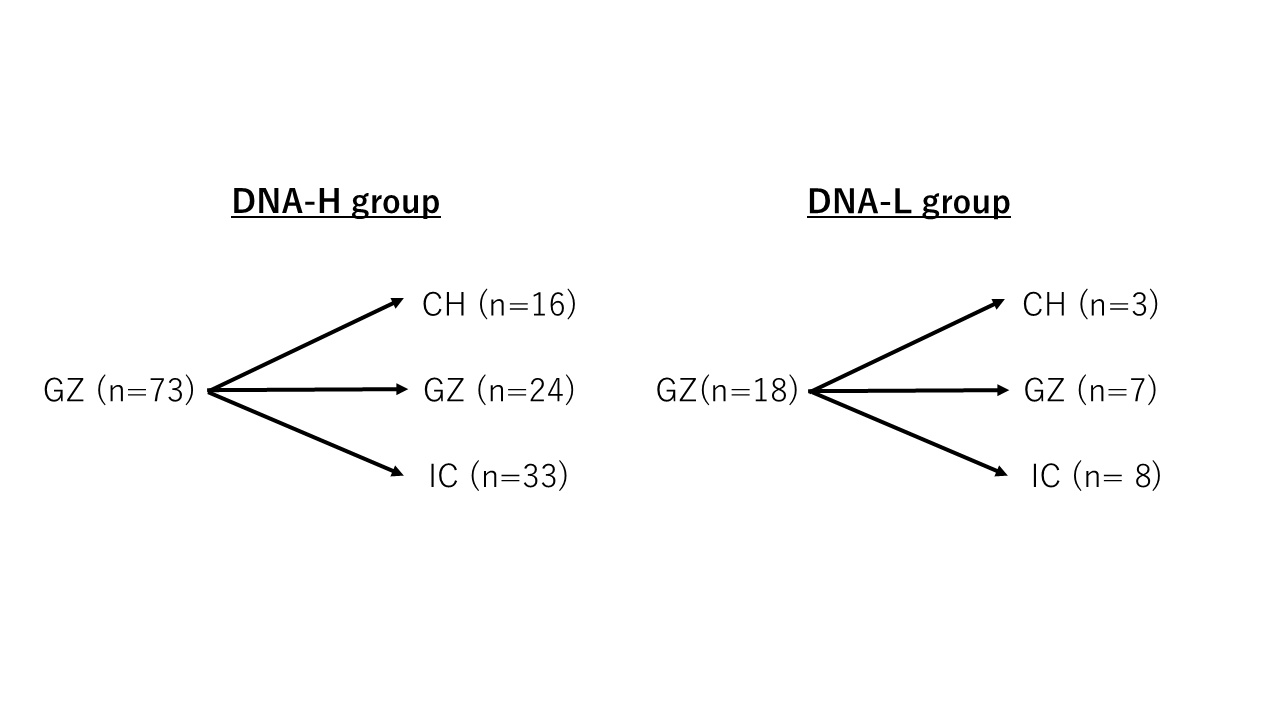


Supplementary Figure 2


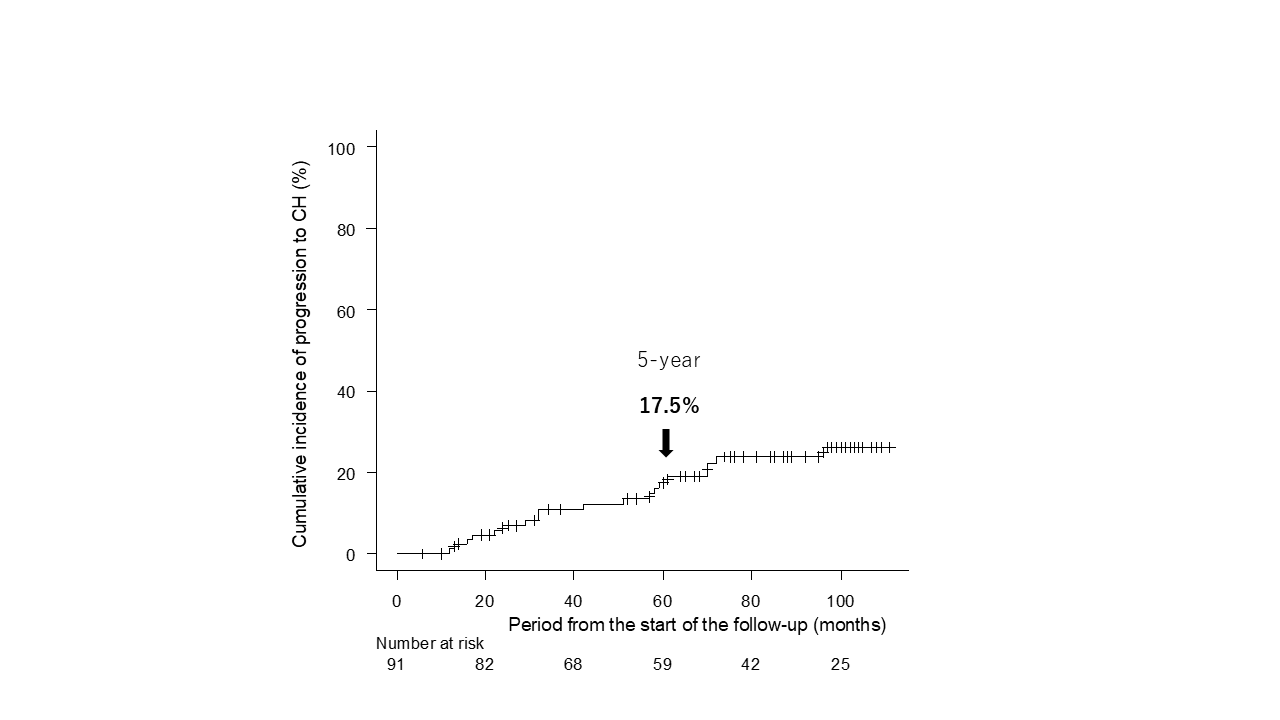


Supplementary Figure 3

**Supplementary Table 1.** **Comparison of baseline characteristics between patients with available and unavailable HBcrAg**

| **Number (n = 102)** | **Available HBcrAg (n = 76)** | **Unavailable HBcrAg (n = 26)** | ***P*-value** |
| --- | --- | --- | --- |
| **DNA-H /DNA-L group** | **60 / 16** | **21 / 5** | **1.000** |
| **Age, years** | **48 (40‒58)** | **54 (47‒61)** | **0.088** |
| **Gender, male / female** | **35 / 41** | **6 / 20** | **0.063** |
| **Hypertension, yes / no** | **10 / 66** | **1 / 25** | **0.281** |
| **Diabetes, yes / no** | **13 / 63** | **4 / 22** | **1.000** |
| **Hyperlipidemia, yes / no** | **10 / 66** | **4 / 22** | **0.750** |
| **Platelet count (× 10^4^/μL)** | **20.7 (17.9‒23.9)** | **23.1 (20.6‒26.7)** | **0.034*** |
| **AST (U/L)** | **22 (19‒30)** | **23 (19‒27)** | **0.911** |
| **ALT (U/L)** | **20 (16‒30)** | **20 (14‒27)** | **0.633** |
| **Albumin (mg/dL)** | **4.5 (4.3‒4.7)** | **4.5 (4.2‒4.6)** | **0.395** |
| **Total bilirubin (mg/dL)** | **0.8 (0.6‒1.0)** | **0.9 (0.6‒1.0)** | **0.613** |
| **FIB-4 index** | **1.23 (0.87‒1.63)** | **1.18 (0.90‒1.51)** | **0.839** |
| **HBV DNA level (log IU/mL)** | **3.6 (3.3‒4.3)** | **3.6 (3.4‒4.3)** | **0.655** |
| **HBsAg level (IU/mL)** | **988.79 (237.16‒2897.08)** | **705.15 (123.05‒1847.69)** | **0.245** |
| **Time from TE to the last visit, months** | **87 (61‒101)** | **67 (17‒101)** | **0.209** |
| **TE, kPa** | **4.4 (3.4‒5.3)** | **3.5 (3.3‒4.5)** | **0.104** |
| **CAP, dm/mm** | **216 (186‒246)** | **223 (194‒252)** | **0.386** |

DNA-H group: HBeAg-negative GZ patients who had high HBV DNA levels ≥3.3 log IU/mL and ALT <31 U/L.

DNA-L group: HBeAg-negative GZ patients who had low HBV DNA levels <3.3 log IU/mL and ALT ≥31 U/L.

Data are expressed as numbers for categorical data and medians (first–third quartiles) for non-categorical data, and compared using the chi-square test and the Mann–Whitney *U* test, respectively.

*, *P*<0.05; **, *P*<0.005.

Abbreviations: HBeAg, hepatitis B e antigen; GZ, gray zone; AST, aspartate aminotransferase; ALT, alanine aminotransferase; FIB-4, fibrosis-4; HBV, hepatitis B virus; HBcrAg, hepatitis B core related antigen; HBsAg, hepatitis B surface antigen; TE, transient elastography; CAP, controlled attenuation parameter.
