# Supplementary material for: Hepatitis B Core‐Related Antigen Level Predicts Disease Progression in the Gray Zone Hepatitis B Patients
Source: J Med Virol. 2026 Apr 8;98(4):e70925. doi: 10.1002/jmv.70925 (PMC13060282; doi:10.1002/jmv.70925)
Supplement: Supplementary file 2 — Supporting File 2 [file JMV-98-e70925-s002.docx]

Figurementary Figure 1. Transition of disease stages in HBeAg-negative GZ patient.

DNA-H group: HBeAg-negative GZ patients who had high HBV DNA levels ≥3.3▒log IU/mL and ALT▒<▒31▒U/L. DNA-L group: HBeAg-negative GZ patients who had low HBV DNA levels <3.3▒log IU/mL and ALT▒≥▒31▒U/L.

Abbreviations: HBeAg, hepatitis B e antigen; GZ, gray zone; CH, chronic hepatitis; IC, inactive carrier.

Figurementary Figure 2. Cumulative incidence rates of progression to CH of HBeAg-negative GZ patients.

Abbreviations: CH, chronic hepatitis; HBeAg, hepatitis B e antigen; GZ, gray zone.

Figurementary Figure 3. Receiver operating characteristic curve analysis to assess the effectiveness of HBcrAg levels for predicting the progression to CH of HBeAg-negative GZ patients.

Abbreviations: HBcrAg, hepatitis B core-related antigen; CH, chronic hepatitis; HBeAg, hepatitis B e antigen; GZ, gray zone; IC, inactive carrier; AUC, area under the curve; 95% CI, 95% confidence intervals.
